# Transcriptomic Analysis of Mouse Brain After Traumatic Brain Injury Reveals That the Angiotensin Receptor Blocker Candesartan Acts Through Novel Pathways

**DOI:** 10.3389/fnins.2021.636259

**Published:** 2021-03-22

**Authors:** Peter J. Attilio, Dustin M. Snapper, Milan Rusnak, Akira Isaac, Anthony R. Soltis, Matthew D. Wilkerson, Clifton L. Dalgard, Aviva J. Symes

**Affiliations:** ^1^Graduate Program in Neuroscience, Uniformed Services University of the Health Sciences, Bethesda, MD, United States; ^2^Department of Pharmacology and Molecular Therapeutics, Uniformed Services University of the Health Sciences, Bethesda, MD, United States; ^3^The American Genome Center, Uniformed Services University of the Health Sciences, Bethesda, MD, United States; ^4^Department of Anatomy, Physiology and Genetics, Uniformed Services University of the Health Sciences, Bethesda, MD, United States

**Keywords:** traumatic brain injury, angiotensin, RNA seq, hippocampus, candesartan, transcriptomic (RNA-Seq)

## Abstract

Traumatic brain injury (TBI) results in complex pathological reactions, where the initial lesion is followed by secondary inflammation and edema. Our laboratory and others have reported that angiotensin receptor blockers (ARBs) have efficacy in improving recovery from traumatic brain injury in mice. Treatment of mice with a subhypotensive dose of the ARB candesartan results in improved functional recovery, and reduced pathology (lesion volume, inflammation and gliosis). In order to gain a better understanding of the molecular mechanisms through which candesartan improves recovery after controlled cortical impact injury (CCI), we performed transcriptomic profiling on brain regions after injury and drug treatment. We examined RNA expression in the ipsilateral hippocampus, thalamus and hypothalamus at 3 or 29 days post injury (dpi) treated with either candesartan (0.1 mg/kg) or vehicle. RNA was isolated and analyzed by bulk mRNA-seq. Gene expression in injured and/or candesartan treated brain region was compared to that in sham vehicle treated mice in the same brain region to identify genes that were differentially expressed (DEGs) between groups. The most DEGs were expressed in the hippocampus at 3 dpi, and the number of DEGs reduced with distance and time from the lesion. Among pathways that were differentially expressed at 3 dpi after CCI, candesartan treatment altered genes involved in angiogenesis, interferon signaling, extracellular matrix regulation including integrins and chromosome maintenance and DNA replication. At 29 dpi, candesartan treatment reduced the expression of genes involved in the inflammatory response. Some changes in gene expression were confirmed in a separate cohort of animals by qPCR. Fewer DEGs were found in the thalamus, and only one in the hypothalamus at 3 dpi. Additionally, in the hippocampi of sham injured mice, 3 days of candesartan treatment led to the differential expression of 384 genes showing that candesartan in the absence of injury had a powerful impact on gene expression specifically in the hippocampus. Our results suggest that candesartan has broad actions in the brain after injury and affects different processes at acute and chronic times after injury. These data should assist in elucidating the beneficial effect of candesartan on recovery from TBI.

## Introduction

Traumatic brain injury (TBI) can result in permanent difficulties with sleep, concentration, memory and mood, even from a seemingly minor injury (Katz et al., 2015; Cole and Bailie, 2016; Giza et al., 2018; Paredes et al., 2020). Morbidity increases with the severity of injury (LoBue et al., 2019; Svingos et al., 2019). Damage from the initial impact, the primary injury, occurs even without external signs, or abnormal CT scans in patients (Schweitzer et al., 2019; Lefevre-Dognin et al., 2020). However, the primary lesion may cause significant internal injuries including axonal shearing and blood brain barrier damage (Laskowski et al., 2015; Ichkova et al., 2017). This injury also initiates secondary cascades of inflammation, oxidative stress, apoptosis and excitotoxicity that result in glial reactivity, further axonal damage, activation of the innate and acquired immune systems, dysfunction of neuronal circuitry, and interruption of cerebrovascular flow (Ling et al., 2015; Ichkova et al., 2017; Sullan et al., 2018; Sta Maria et al., 2019). These secondary cascades worsen the initial impact and provide a therapeutic window to intervene in order to reduce pathology and improve recovery (Diaz-Arrastia et al., 2014). However, despite many years of research, and over 30 clinical trials, there is no FDA approved therapy to treat TBI (Armstead and Vavilala, 2020; Figaji et al., 2017; Xiong et al., 2015). TBI impacts all cell types in the area of injury, so therapies that are broadly acting may have greater potential to succeed.

The renin angiotensin system (RAS) is predominantly recognized as a major regulator of systemic blood pressure and fluid homeostasis (Miller and Arnold, 2019; Mirabito Colafella et al., 2019). However, the brain expresses all components of the RAS, and it is not acknowledged that the brain RAS influences many aspects of brain function that are independent of the regulation of systemic blood pressure, including the stress response, limbic function, sensory responses, regulation of cerebral circulation and sympathetic activity (Wright and Harding, 2004; Krause et al., 2011; Bali and Jaggi, 2013; Kangussu et al., 2013; Marvar et al., 2014; Hurt et al., 2015; Wang et al., 2016a; Miller and Arnold, 2019; Yu et al., 2019). Angiotensin II, acting through the AT1 Receptor in neurons, astrocytes, microglia and endothelial cells, stimulates oxidative stress, inflammatory signaling, apoptosis and vasoconstriction (Saavedra, 2005; Saavedra et al., 2006; Pavel et al., 2008; Benicky et al., 2009; Villapol and Saavedra, 2015). As there are many similarities between some of the cascades activated after traumatic brain injury, and those initiated by AT1R signaling, it was proposed that AT1R signaling in different cell types could contribute to some of the adverse reactions after TBI (Thone-Reineke et al., 2006; Timaru-Kast et al., 2012). In support of this, mice that lack the AT1R have a smaller lesion after controlled cortical impact injury (Villapol et al., 2015). Further, drugs that block signaling through the AT1R can improve recovery after TBI in the mouse (Timaru-Kast et al., 2012; Villapol et al., 2012; Villapol et al., 2015; Janatpour and Symes, 2020). These drugs, called angiotensin receptor blockers (ARBs) have also been shown to be neuroprotective in rodent models of other disorders of the CNS including Parkinson’s disease, Alzheimer’s disease, stroke, cerebral hemorrhage and cerebral edema initiated by systemic inflammation (Kehoe, 2009; Villapol and Saavedra, 2015; Saavedra, 2017). Clinical trials have shown reduced incidence of stroke in patients on ARBs (Thone-Reineke et al., 2006; Sandset et al., 2010; Regenhardt et al., 2014). ARBs have also been shown to reduce the progression from mild cognitive impairment towards Alzheimer’s disease, and to reduce the incidence of Alzheimer’s disease in patients on long term ARB therapy for hypertension (Davies et al., 2011; Zhuang et al., 2016; Ho and Nation, 2017; Kehoe, 2018). This seems to be distinct from the general protective effects of controlling hypertension (Saavedra, 2016). Further, retrospective studies have shown that ARB therapy leads to lower symptoms of post-traumatic stress disorder (PTSD), and reduced markers for depression in specific populations (Khoury et al., 2012; Boal et al., 2016; Hurt et al., 2015). Patients with TBI often have overlapping symptoms with PTSD, including depression and sleep disruptions (Stein and McAllister, 2009; Howlett and Stein, 2016), and TBI increases the overall risk of Alzheimer’s disease (Chauhan, 2014; Crane et al., 2016; LoBue et al., 2019). Thus, treatment of TBI with ARBs has the potential to reduce both the acute and chronic consequences of the injury.

Many of the neuroprotective effects of ARBs have been attributed to their anti-inflammatory effects in CNS tissues acting through both AT1R dependent and independent mechanisms (Benicky et al., 2011; Saavedra, 2011; Villapol et al., 2015). A major AT1R independent effect of ARBs is the ability of some specific ARBs to act as PPARγ partial agonists (Schupp et al., 2004; Miura et al., 2011). Telmisartan, candesartan, losartan and irbesartan have this dual ability to differing effects (Benson et al., 2004; Schupp et al., 2004; An et al., 2010). Stimulating PPARγ in addition to AT1R antagonism provides a dual mechanism to act in an anti-inflammatory manner and broadens the range of cells that ARBs will reach to improve recovery. Indeed, PPARγ activity is prominent in microglia and oligodendrocytes whereas there is some dispute about AT1R signaling in these cells *in vivo* (Bernardo and Minghetti, 2006; Kapadia et al., 2008; McCarthy et al., 2013; Wu et al., 2013; Xu et al., 2015). Additionally, some ARBs also can activate AMPK signaling within microglia, and potentially have other effects on different signaling pathways (Xu et al., 2015). We have previously shown that the ARB, candesartan, can improve behavioral and pathologic recovery after controlled cortical impact injury in mice (Villapol et al., 2012, 2015). Candesartan, administered 6 hours after injury, increases cognitive function four weeks after injury, reduces lesion volume, and reduces astrocyte and microglial activation (Villapol et al., 2012, 2015).

In order to understand better the mechanisms through which candesartan acts after TBI, we have performed RNA seq analysis on different brain regions at 3 and 29 days post injury in order to determine how candesartan treatment alters the response to injury. We examined gene expression in the hippocampus, the region immediately under the lesion, the thalamus where the cortical thalamic neurons have their cell bodies and the hypothalamus, the site of the most dense expression of AT1R in the brain (Lenkei et al., 1997). We used a low subhyptotensive dose of candesartan, that we and others have previously shown to enhance recovery in this mouse model of TBI (Timaru-Kast et al., 2012; Villapol et al., 2012, 2015). We found that the clearest gene expression differences were found between different brain regions independent of injury status. Injury had stronger effects at a more acute time point [3 days post injury (dpi)], and the effects of candesartan were more muted. Nevertheless, we were able to determine that candesartan influenced numerous pathways after injury in mice at both 3 and 29 dpi, with the largest effects found in the hippocampus. Understanding the molecular mechanisms of these treatments in animal models may assist in determining pathways that are critical to drug efficacy and provide biomarkers of drug target engagement.

## Materials and Methods

### Animals

All animal studies were approved by the Uniformed Services University of the Health Sciences (USUHS) Institutional Animal Care and Use Committee and were conducted in accordance with the NRC guide to the Care and Use of Laboratory Animals. Adult male age-matched (8-10 week old) C57BL/6 mice weighing 20–25g were obtained from Charles River Laboratories (Frederick, MD, United States). All mice were kept under 12:12 h light and dark cycle with access to food and water *ad libitum*. Five mice were housed in each cage. After arrival, mice were acclimatized for one week before use. Animals were randomly assigned to receive a controlled cortical impact (CCI) injury or sham injury as well as treatment with candesartan or vehicle.

### Controlled Cortical Impact Injury

Animals in the injury group were given a moderate brain injury utilizing a CCI. The CCI was performed on the animals as previously described (Villapol et al., 2012). In brief, animals were anesthetized with isoflurane (3% induction, 2% maintenance) and placed into a stereotactic mount. The animal’s scalp was shaved and the head secured with ear bars. A 3 mm craniectomy was made (2 mm lateral (left), 2 mm caudal to bregma) over the location of the impact site. A pneumatic impactor (Impact One stereotaxic impactor Leica Microsystems, Buffalo Grove, IL, United States) with a 2mm rounded impact tip was used to deliver the CCI (3.6 m/s, 1.5 mm depth, 100 ms dwell time, 12° angle to the dura mater). The scalp was then sutured closed and the animal allowed to recover prior to placing back in the cage. Sham animals received the same exposure to anesthesia and scalp excision, but without a craniectomy or injury.

### Drug Treatment

Candesartan can cross the blood brain barrier (Nishimura et al., 2000) so we administered it peripherally either through intraperitoneal (i.p.) injection (3 day cohort), or osmotic minipump (29 day cohort). Candesartan was resuspended in 0.1N Na_2_CO_3,_ pH 7.4, and administered at 0.1 mg/kg/day. All mice received their first dose of either candesartan (Tocris Bioscience, Minneapolis, MN, United States # 4791) or vehicle (0.9% saline and 0.1N Na_2_CO_3_ at pH = 7.4) six hours after the CCI or sham procedure through i.p. injection. Mice in the 3 day cohort received two subsequent i.p. injections at 24- and 48-h after CCI, before sacrifice at 3 dpi. Mice in the 29-day cohort were surgically implanted with an osmotic pump (#1004, Alzet, Cupertino, CA, United States) 24 h after the CCI or sham procedure. Pumps were placed in the lower back through a tunneled incision at the base of the neck under isoflurane anesthesia. Prior to implantation the osmotic pumps were primed with candesartan or vehicle at 37°C overnight.

### Brain Region Isolation and RNA Extraction

At either 3 or 29 dpi, mice were anesthetized with ketamine and xylazine and perfused with ice-cold filtered 0.9% saline solution and decapitated. Brains were removed and individual brain regions dissected. Tissue was placed into TRI reagent (Zymo Scientific, Tustin, CA, United States # R2050-1-50) and triturated through a 22G needle followed by a 25G needle. RNA was extracted utilizing the Direct-zol RNA MiniPrep kits (Zymo Research, # R2025) according to the manufacturer’s instructions. RNA was quantified by UV spectrometry with a NanoDrop^TM^ UV-Vis Spectrophotometer (Thermo Scientific, United States). RNA quality for the RNA sequencing was assessed using the BioRad Experion Automated Electrophoresis System (BioRad, Hercules, CA, United States). RNA quality for qPCR was checked by agarose gel electrophoresis.

### RNA Sequencing

The four highest quality RNA samples per group were identified, aliquoted, given unique identifiers, and sent to the Collaborative Health Initiative Research Program (CHIRP) American Genome Center at USUHS for processing. Total RNA integrity was assessed using automated capillary electrophoresis on a Fragment Analyzer (Roche). For all samples RQI > 8.0, a total RNA amount of > 75 ng was used as input for library preparation using the TruSeq Stranded mRNA Library Preparation Kit (Illumina, San Diego, CA, United States). Sequencing libraries were quantified by PCR using KAPA Library Quantification Kit for NGS (Kapa, Wilmington, MA, United States) and assessed for size distribution on a Fragment Analyzer. Sequencing libraries were pooled and sequenced on a HiSeq 3000 System (Illumina) using a HiSeq 3000/4000 PE Cluster Kit and SBS Kit (150 cycles) with run conditions of paired-end reads at 75 bp length. Raw sequencing data were demuxed using bcl2fastq2 conversion software 2.20.

#### Bioinformatics Analysis

RNA-seq samples were aligned to the mouse genome (mm10) using MapSplice (v. 2.2.1), expression quantification of individual genes was performed using HTSeq (v. 0.9.1), and differential gene expression was performed using DESeq2 (v 1.16.1). Individual groups were compared to sham mice and differentially expressed genes (DEGs) were identified with a false discovery rate of 0.05 and an absolute log2 fold-change (abs log2 FC) > 0.32.

### Unique Gene ID and Gene Ontology Analysis

Differentially expressed genes that were unique to either the TBI-candesartan or the TBI-vehicle group at each time point within the hippocampal data were identified for both up-regulated and down-regulated DEGs. Gene Ontology (GO) analysis was performed on unique up- and down-regulated DEGs altered by candesartan and vehicle after TBI at 3 dpi and 29 dpi in the hippocampus, using the PANTHER Overrepresentation Test (Release 20190429) with the Mus musculus GO database (Released 2020-06-01^1^). GO biological process terms were identified using a Fisher’s Exact test and a false discovery rate of 0.05 (Ashburner et al., 2000; Mi et al., 2017).

### Pathway Enrichment Analysis

Pathway enrichment analysis was performed on unique up- and down-regulated DEGs altered by candesartan and vehicle after TBI at 3 dpi in the hippocampus, using Molecular Signatures Database v7.1 (MSigDB v7.1^2^) (Subramanian et al., 2005; Liberzon et al., 2015). DEGs were converted to Mouse Ensemble IDs (species: Mus musculus), which were used as the input gene list. The analyses included the selected canonical pathway databases: BIOCARTA^3^, KEGG^4^, PID^5^, and REACTOME^6^. Pathways were identified using a Fisher’s exact test with a false discovery rate of less than 0.05. A minimum gene set size of 5, and a maximum gene set size of 350 were used as additional filters.

### Functional Interaction Network

A functional interaction network was created using Cytoscape (v3.8.0) with the ReactomeFIViz plugin (v.7.2.3) (Shannon et al., 2003; Wu and Haw, 2017). To create the network, the up-regulated DEGs in the TBI candesartan group at 3 dpi in the hippocampus were used as input for the gene set analysis feature (Reactome FI Network Version 2019). Unlinked genes were not included in the analysis. The network was then clustered into functional modules and were further analyzed for pathway enrichment using a false discovery rate of less than 0.05.

### qPCR

cDNA was synthesized using SuperScript III (Life Technologies) and qPCR performed with PerFecTa SYBR Green mastermix (QuantaBio, Beverly, MA, United States) in a CFX96 Thermocycler (Bio-Rad). The following primers were used: Vim - forward 5′-GCCAGATGCGTGAGATGGA-3′, reverse 5′-GGC GATCTCAATGTCCAGGG-3′; Lyz2 (lysozyme C-2 precursor) - forward 5′-AGCACTGTACCCCACCATTT-3′, reverse 5′-CTT TTCCTTCTCAGGGGTGTG-3′, Gpnmb (glycoprotein NMB) - forward 5′-CTGCTTTAAAGACCCAGACTCC-3′, reverse 5′-ACTTACTTGTACAGCAAGATGGTAA-3′; C4b (complement C4B) - forward 5′-ACTTACTTGTACAGCAAGATGGTAA-3′, reverse 5′-ACACTGTGCTCTGGAGATGT-3′, Ywhaz forward 5′-CTTTCTGGTTGCGAAGCATT-3′, reverse 5′-TTGAGCAG AAGACGGAAGGT-3′; Rpl13 forward 5′-CTTTTCCCAG ACGAGGATATTCC-3′, reverse 5′-CCAGCCGTTTAGGCA CTCT-3′. Target gene expression was normalized to the housekeeping genes Rpl13 or Ywhaz using the delta threshold cycle (ΔΔCt) method (Susarla et al., 2011) and analyzed with Bio-Rad CFX Manager 2.0 software.

### Statistical Analysis for qPCR

All data are expressed as mean + SEM. Statistical analysis was performed utilizing Prism software (version 8.1). Relative gene expression determined by qPCR was compared between sham and injured groups, and between vehicle and drug treated groups using a two-way ANOVA with Holm’s Sidak multiple comparisons correction. *P* < 0.05 was considered statistically significant.

## Results

**FIGURE 1:**
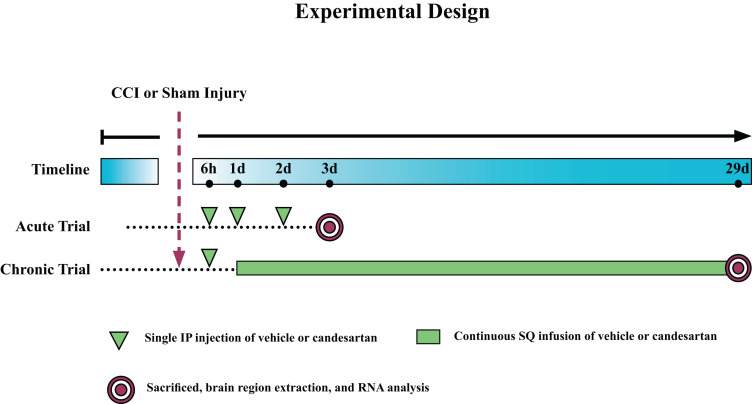
Experimental Design. Mice were subjected to controlled cortical impact (CCI) or sham injury and sacrificed at either 3 days post-injury (dpi) (acute) or 29 dpi (chronic). Mice in the acute trial received three 0.1 mg/kg intraperitoneal (IP) injections of candesartan or vehicle at 6, 24, and 48 h post-injury. Mice in the chronic trial received an initial dose of 0.1 mg/kg candesartan or vehicle at 6 h post-injury and then at 1 dpi, were implanted with osmotic pumps with continuous SQ infusion of candesartan or vehicle (0.1 mg/kg/day) until sacrifice. Brains were removed and the hippocampus, thalamus, and hypothalamus dissected, and RNA isolated from these regions.

We determined gene expression in three different brain regions of mice at either 3 days or 29 days after controlled cortical impact or sham injury. Mice were treated either with candesartan (0.1 mg/kg/day) or vehicle, starting 6 hours after injury (Figure 1). Gene expression was determined in the ipsilateral hippocampus, thalamus or hypothalamus by bulk mRNA seq analysis.

### Semi-Supervised Hierarchical Clustering and Principal Component Analysis

**FIGURE 2:**
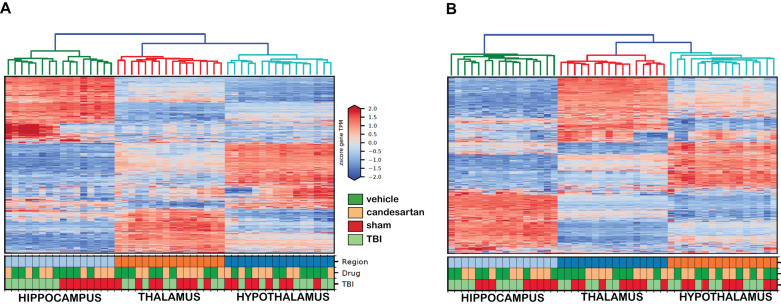
Semi-Supervised Hierarchical Clustering of Genes at 3 and 29 dpi. The top 5,000 genes according to median absolute deviation (MAD) of the gene transcripts per million (TPM) across all samples underwent semi-supervised hierarchical clustering (*n* = 4/group). Samples clustered primarily by brain region at 3 dpi **(A)** and at 29 dpi **(B)**.

**FIGURE 3:**
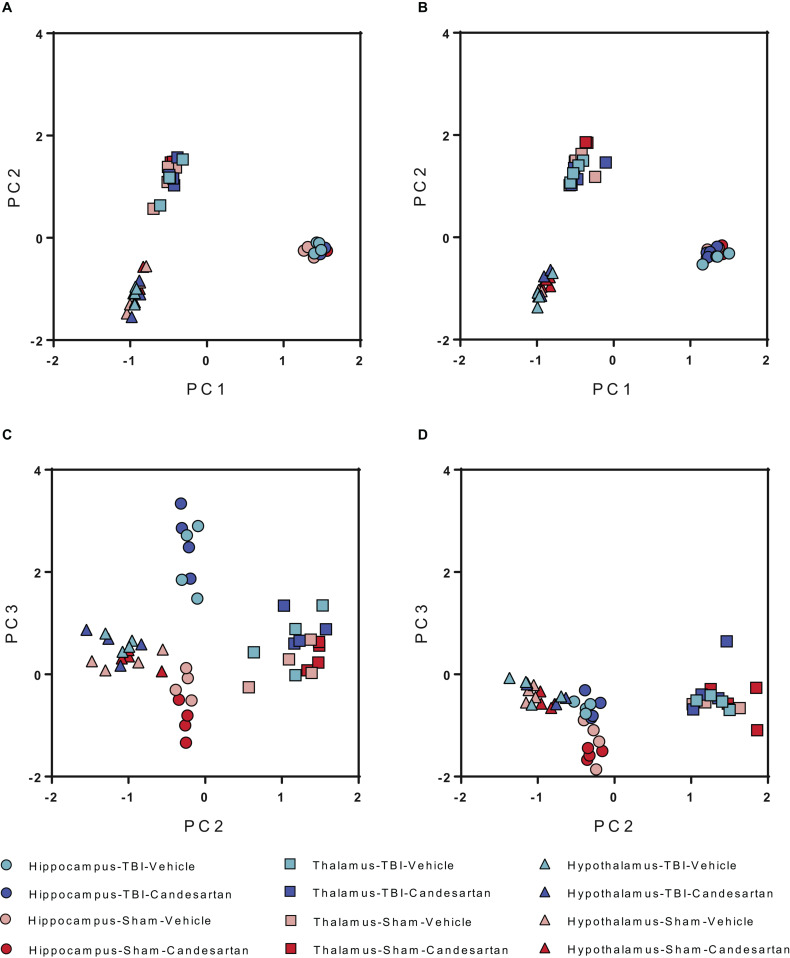
Principal Component Analysis (PCA) of Differentially Expressed Genes at 3 and 29 dpi. PCA analysis shows the largest differences between brain regions at both **(A)** 3 dpi and **(B)** 29 dpi for all conditions. Differences are also seen between TBI and sham in the hippocampus at **(C)** 3 dpi but not at **(D)** 29 dpi (*n* = 4/group).

After quality analysis of samples, the genes were mapped utilizing semi-supervised hierarchical cluster according to the median absolute deviation (MAD) of the gene transcripts per million (TPM) (Figure 2) for both 3 and 29 dpi cohorts. The hierarchical clustering showed the greatest differentiation among the samples by brain region at either time point. The second greatest differentiation in clustering occurred only within the hippocampus in the 3 dpi samples and showed differentiation between RNA taken from TBI and Sham mouse brains. We did not observe defined clustering according to candesartan treatment in either time point or in any brain region. These observations were confirmed by principal component analysis (PCA) (Figure 3). The largest observed variation was between samples taken from the different brain regions regardless of injury status or candesartan treatment at either time point (Figures 3A,B). The second largest variation was observed between the samples taken from the hippocampus at 3 dpi after TBI and sham injury. This difference had almost disappeared at 29 dpi in the hippocampal samples and was not detectable in the samples taken from the thalamus or hypothalamus at either 3 or 29 dpi.

### Differential Gene Expression

Differential gene expression analysis was performed within each brain region at each time point, comparing the gene expression after TBI +/− candesartan treatment to that in the sham injury and vehicle treated group (Sham + VH). As the intent of the project was to identify changes in gene expression due to candesartan treatment, we chose to identify DEGs with a log2 > 0.32 as larger cut offs were associated only with changes caused by TBI. The number of differentially expressed genes (DEGs) for each condition are listed in Table 1.

**TABLE 1:** Total Differentially Expressed Genes.

**3DPI**	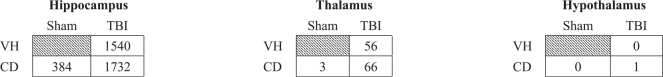
**29DPI**	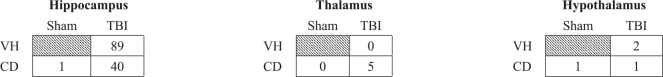

*Total number of differential expressed genes found in the hippocampus, thalamus, and hypothalamus after TBI +/− candesartan (CD) treatment in comparison with genes expressed in those regions in sham + vehicle (VH) mice at 3 and 29 dpi. Differentially expressed genes (DEGs) were identified with a false discovery rate of 0.05 and an absolute log2 fold change > 0.32. VH, vehicle; CD, candesartan.*

The largest number of DEGs were found in the hippocampus at 3 dpi. This region is closest to the cortical lesion and therefore the most altered by the injury. At 3 dpi, the hippocampus showed a large effect in both TBI groups with 1732 DEGs identified in the TBI with candesartan treated group (TBI + CD) and 1540 DEGs identified in the TBI and vehicle treated group (TBI + VH) (Supplementary Table 1). A TBI effect was identifiable within the thalamus but fewer DEGs were noted compared to those in the hippocampus. This included 56 DEGs in the TBI + VH group and 66 DEGs with candesartan treatment (TBI + CD) (Supplementary Table 1). At 29 dpi, the largest number of DEGs was again observed in the hippocampus. However, their number was far smaller than at 3 dpi with 89 DEGs in vehicle alone (TBI + VH) and 40 DEGs with candesartan treatment (TBI + CD). The effect of TBI in the thalamus at 29 dpi had nearly completely dissipated and the hypothalamus showed little effect at either 3 or 29 dpi.

Interestingly, at 3 dpi, there were a large number of DEGs identified in the hippocampus of mice after candesartan treatment in the sham group (Sham + CD) compared to the Sham + VH group. A total of 384 genes were differentially expressed in response to candesartan treatment in the sham mice at this time point. 358 DEGs were down-regulated whereas only 26 were up-regulated (Supplementary Table 1). GO analysis of the down-regulated unique DEGs identified common GO terms related to cilium organization, cilium assembly, and cell adhesion (Supplementary Table 2). GO analysis within the up-regulated unique DEGs identified GO terms associated with voltage gated cation channel activity (Supplementary Table 3). The thalamus and the hypothalamus had few DEGs with candesartan treatment in sham mice. By 29 dpi there was only 1 DEG identified in the hippocampus and hypothalamus with candesartan treatment and sham injury.

### Unique Gene Analysis

**FIGURE 4:**
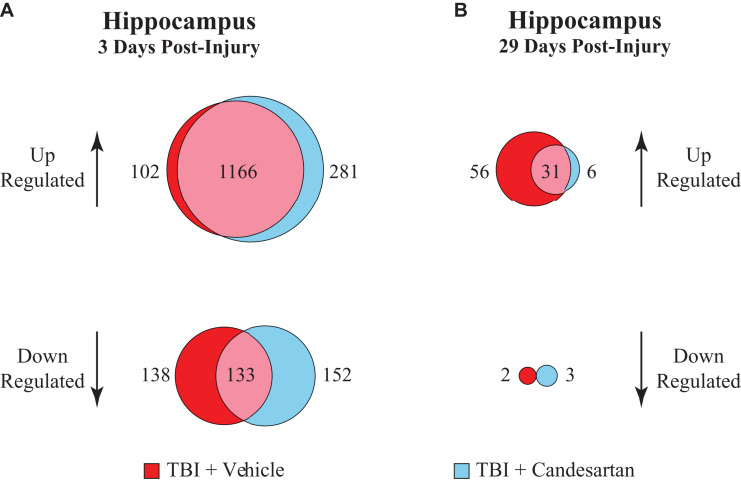
Unique gene analysis in the hippocampus at 3 and 29 dpi. Differentially expressed genes (DEGs) that were either upregulated or down-regulated after TBI relative to those in the sham + vehicle group, were compared between vehicle and candesartan treatments at **(A)** 3 and **(B)** 29 dpi in the hippocampus. DEGs were identified with a false discovery rate of 0.05 and an absolute log2 fold-change (abs log2 FC) > 0.32.

A direct statistical comparison between the transcriptomes of the TBI + CD and the TBI + VH mouse hippocampi only produced one differentially expressed gene (Kcnh7), presumably because of the large effect of TBI on gene expression. Therefore, to identify DEGs that were only found in either the TBI + CD or the TBI + VH group, we carried out a unique gene analysis and performed GO analysis on these “unique DEGs.” Analysis was focused on the DEGs found in the hippocampus as this was the brain region with the most changes in gene expression. DEGs that were shared in both conditions were attributed to a common TBI effect (1166 DEGs). The remaining genes were determined to be the unique up- and down-regulated genes within that condition (Figure 4).

Analysis of the DEGs that were up-regulated relative to Sham + VH at 3 dpi in the hippocampus, showed that there were 281 DEGs unique to the TBI + CD group, and 102 DEGs unique to the TBI + VH group (Figure 4). Gene Ontology (GO) analysis of the unique up-regulated genes in the TBI + CD group identified GO terms associated with the regulation of stress, stress responses and wounding, as well as cell adhesion (Table 2). GO analysis of unique up-regulated genes in the TBI + VH group identified no GO terms.

**TABLE 2:** Gene Ontology Analysis of Unique Genes at 3 dpi in the Hippocampus.

**Up or Down**	**Treatment**	**GO Biological Process**	**FDR**
Up-Regulated	Candesartan	Response to Stress (GO:0006950)	1.23E-05
		Regulation of Cell Adhesion (GO:0030155)	6.44E-05
		Response to Chemical (GO:0042221)	9.95E-04
		Positive Regulation of Cell Adhesion (GO:0045785)	1.05E-03
		Cellular Response to Nitrogen Compound (GO:1901699)	1.18E-03
		Cellular Response to Chemical Stimulus (GO:0070887)	1.34E-03
		Response to Wounding (GO:0009611)	1.38E-03
		Cellular Response to Stress (GO:0033554)	2.36E-03
		Response to Stimulus (GO:0050896)	2.98E-03
		Circulatory System Development (GO:0072359)	4.79E-03
		Tube Development (GO:0035295)	4.85E-03
		Wound Healing (GO:0042060)	4.91E-03
		Positive Regulation of Biological Process (GO:0048518)	5.16E-03
		Regulation of Programmed Cell Death (GO:0043067)	5.22E-03
		Regulation of Apoptotic Process (GO:0042981)	7.02E-03
		Response to Nitrogen Compound (GO:1901698)	7.67E-03
		Blood Vessel Development (GO:0001568)	7.84E-03
		Positive Regulation of Cellular Process (GO:0048522)	7.99E-03
		Metabolic Process (GO:0008152)	8.11E-03
		Regulation of Developmental Process (GO:0050793)	8.40E-03
	Vehicle	No GO Families Identified	
Down-Regulated	Candesartan	Cilium Assembly (GO:0060271)	3.46E-07
		Cilium Organization (GO:0044782)	3.83E-07
		Microtubule Bundle Formation (GO:0001578)	4.08E-07
		Axoneme Assembly (GO:0035082)	7.29E-07
		Cilium Movement (GO:0003341)	3.29E-06
		Plasma Membrane Bounded Cell Projection Assembly (GO:0120031)	1.30E-05
		Cell Projection Assembly (GO:0030031)	1.96E-05
		Organelle Assembly (GO:0070925)	1.79E-04
		Microtubule-Based Process (GO:0007017)	2.88E-04
		Cell Projection Organization (GO:0030030)	1.42E-03
		Plasma Membrane Bounded Cell Projection Organization (GO:0120036)	2.20E-03
		Microtubule Cytoskeleton Organization (GO:0000226)	2.74E-03
		Microtubule-Based Movement (GO:0007018)	6.10E-03
		Cilium or Flagellum-Dependent Cell Motility (GO:0001539)	6.53E-03
		Cilium-Dependent Cell Motility (GO:0060285)	6.99E-03
		Cytoskeleton Organization (GO:0007010)	7.02E-03
		Sperm Motility (GO:0097722)	3.19E-02
	Vehicle	Anion Transport (GO:0006820)	2.76E-02
		Reproduction (GO:0000003)	4.29E-02
		Organic Anion Transport (GO:0015711)	4.48E-02
		Reproductive Process (GO:0022414)	5.31E-02
		Multicellular Organismal Homeostasis (GO:0048871)	6.27E-02

*Gene ontology analysis (biological process) of genes altered by candesartan or vehicle treatment after TBI at 3 dpi in the hippocampus after unique gene analysis. GO biological process terms were identified using a Fisher’s Exact test and false discovery rate of 0.05.*

Unique gene analysis of DEGs that were down-regulated relative to Sham + VH showed that there were 152 DEGs unique to the TBI + CD group and 138 downregulated DEGs unique to the TBI + VH group (Figure 4 and Supplementary Table 4). GO analysis of the unique down-regulated DEGs in the TBI + CD group identified GO terms associated with cellular structure, in particular related to cilium organization, and assembly and movement. These GO categories were also identified in the down-regulated DEGs in the hippocampus of the Sham + CD mice (Supplementary Table 2), showing similar mechanisms of candesartan action in sham or TBI mice. In comparison, unique down-regulated DEGs in the TBI + VH group showed a weak association with GO terms associated with anion transport and reproductive processes.

At 29 dpi in the hippocampus the number of DEGs identified was much reduced compared to those at 3 dpi (Figure 4). A majority of those were unique up-regulated DEGs within the TBI + VH group (Supplementary Table 4). All other groups had relatively few DEGs altered and no GO terms identified. GO analysis of the unique up-regulated DEGs within the vehicle group identified GO terms associated with responses to stress, as well as immune processes (Table 3).

**TABLE 3:** Gene Ontology Analysis of Unique Genes at 29 dpi in the Hippocampus.

**Up or Down**	**Treatment**	**GO Biological Process**	**FDR**
Up-regulated	Candesartan	No GO Families Identified	
	Vehicle	Response to External Stimulus (GO:0009605)	1.19E-13
		Immune System Process (GO:0002376)	1.21E-13
		Defense Response (GO:0006952)	2.95E-13
		Response to Stress (GO:0006950)	3.45E-11
		Response to External Biotic Stimulus (GO:0043207)	1.60E-10
		Response to Other Organism (GO:0051707)	1.81E-10
		Interspecies Interaction Between Organisms (GO:0044419)	1.91E-10
		Response to Biotic Stimulus (GO:0009607)	2.17E-10
		Regulation of Localization (GO:0032879)	7.18E-10
		Inflammatory Response (GO:0006954)	8.79E-10
		Regulation of Multicellular Organismal Process (GO:0051239)	1.58E-09
		Immune Response (GO:0006955)	3.67E-09
		Response to Chemical (GO:0042221)	4.09E-09
		Regulation of Transport (GO:0051049)	1.34E-08
		Regulation of Immune Response (GO:0050776)	3.10E-08
		Defense Response to Other Organism (GO:0098542)	3.93E-08
		Positive Regulation of Biological Process (GO:0048518)	4.16E-08
		Positive Regulation of Cellular Process (GO:0048522)	1.18E-07
		Regulation of Cytokine Production (GO:0001817)	1.27E-07
		Negative Regulation of Multicellular Organismal Process (GO:0051241)	1.54E-07
Down-regulated	Candesartan	No GO Families Identified	
	Vehicle	No GO Families Identified	

*Gene ontology analysis (biological process) of unique genes altered by candesartan or vehicle treatment after TBI at 29 dpi in the hippocampus after unique gene analysis. GO biological process terms were identified using a Fisher’s Exact test and false discovery rate of 0.05.*

### Pathway Analysis

Out of 281 up-regulated DEGs in the TBI + CD group at 3 dpi in the hippocampus, 261 genes were mapped and 90 significant pathways were identified (FDR < 0.05). The top 20 enriched pathways are presented (Table 4). The enriched pathways were largely involved in the following: (1). DNA replication (chromosome maintenance, lagging strand synthesis, telomere c-strand synthesis, and polymerase switching), (2). extracellular matrix organization (integrin-1 pathway, integrin cell surface interaction, focal adhesions, and integrins in angiogenesis), (3). platelet activation, signaling, and aggregation (response to elevated platelet cytosolic Ca2+), and (4). interferon signaling (interferon alpha/beta signaling). Other pathways such as signaling by moderate kinase activity BRAF mutants and MAP2K and MAPK activation, were also enriched. Out of 152 down-regulated DEGs in the TBI + CD group at 3 dpi in the hippocampus, 139 genes were mapped and no significant pathways were enriched. Out of 102 up-regulated DEGs in the TBI + VH group at 3 dpi in the hippocampus, 97 genes were mapped and no significant pathways were enriched. Out of 139 down-regulated DEGs in the TBI + VH group at 3 dpi in the hippocampus, 131 genes were mapped and 20 significant pathways were identified (FDR < 0.05) (Table 5). The pathways enriched in this group were largely involved in extracellular matrix organization. These pathways include collagen degradation, collagen biosynthesis and modifying enzyme, assembly of collagen fibrils and chain trimerization, laminin interactions, integrin cell surface interaction, and integrins in angiogenesis. Other pathways enriched include response to elevated platelet cytosolic Ca^2+^, SLC-mediated transmembrane transport, cargo concentration in ER, transport of bile salts, organic acids, metal ions and amine compounds, and retinoid cycle disease events. There were three pathways in common between the up-regulated DEGs in the TBI + CD group and down-regulated DEGs in the TBI + VH groups: extracellular matrix organization, integrins in angiogenesis, and response to elevated platelet cytosolic Ca^2+^.

**TABLE 4:** Pathway Analysis of Unique Up-Regulated Genes at 3 dpi in the Hippocampus.

**Pathway Database**	**Pathway Description**	**Genes**	**FDR *q*-value**
Reactome	Extracellular matrix organization	VCAM1, FN1, THBS1, VWF, COL3A1, ITGA1, F11R, COL12A1, ADAM8, MMP2, BMP1, CASP3, FBLN5, LOXL1, BMP4, LRP4	7.41E-07
Reactome	Interferon signaling	VCAM1, FLNA, OAS1, ISG15, USP18, SAMHD1, IFI35, ISG20, BST2, DDX58, TRIM21, SP100, TRIM34	1.74E-06
Reactome	Chromosome maintenance	PCNA, POLA2, PRIM2, RFC3, LIG1, H2BC14, CENPK, CENPO, CENPX	8.60E-05
Reactome	Lagging strand synthesis	PCNA, POLA2, PRIM2, RFC3, LIG1	1.09E-04
Reactome	Response to elevated platelet cytosolic Ca2+	FN1, THBS1, VWF, FLNA, F13A1, VCL, PFN1, PF4, LAMP2	1.25E-04
PID	Integrin-1 pathway	VCAM1, FN1, THBS1, COL3A1, ITGA1, F13A1, MDK	1.25E-04
Reactome	Interferon alpha/beta signaling	OAS1, ISG15, USP18, SAMHD1, IFI35, ISG20, BST2	1.61E-04
Reactome	Telomere c-strand (lagging strand) synthesis	PCNA, POLA2, PRIM2, RFC3, LIG1	3.31E-04
Reactome	Integrin cell surface interactions	VCAM1, FN1, THBS1, VWF, COL3A1, ITGA1, F11R	4.02E-04
Reactome	Polymerase switching	PCNA, POLA2, PRIM2, RFC3	4.02E-04
Reactome	Platelet activation, signaling and aggregation	FN1, THBS1, VWF, FLNA, F13A1, VCL, PFN1, PF4, LAMP2, PIK3R6, GNG10	4.02E-04
Reactome	DNA strand elongation	PCNA, POLA2, PRIM2, RFC3, LIG1	4.43E-04
Reactome	Processive synthesis on the lagging strand	PCNA, POLA2, PRIM2, LIG1	4.53E-04
KEGG	DNA replication	PCNA, POLA2, PRIM2, RFC3, LIG1	6.78E-04
Reactome	Polymerase switching on the C-strand of the telomere	PCNA, POLA2, PRIM2, RFC3	6.78E-04
Reactome	MAP2K and MAPK activation	FN1, VWF, VCL, ACTG1, NRAS	1.04E-03
KEGG	Focal adhesion	FN1, THBS1, VWF, COL3A1, ITGA1, FLNA, VCL, ACTG1, ILK	1.27E-03
PID	Integrins in angiogenesis	FN1, COL3A1, F11R, COL12A1, VCL, ILK	1.41E-03
Reactome	Extension of telomeres	PCNA, POLA2, PRIM2, RFC3, LIG1	1.41E-03
Reactome	Signaling by moderate kinase activity BRAF mutants	FN1, VWF, VCL, ACTG1, NRAS	1.50E-03

*Pathway analysis of unique up-regulated genes altered by candesartan after TBI at 3 dpi in the hippocampus. Pathways were identified using a Fisher’s exact test with a false discovery rate of < 0.05.*

**TABLE 5:** Pathway Analysis of Unique Down-Regulated Genes at 3 dpi in the Hippocampus.

**Pathway Database**	**Pathway Description**	**Genes**	**FDR *q*-value**
Reactome	Core matrisome	COL9A3, COL8A2, COL14A1, COL18A1, HSPG2, FBN1, NID2, PRELP, FNDC1	1.05E-03
Reactome	SLC-mediated transmembrane transport	SLC44A5, SLC31A1, SLC22A8, SLC13A4, SLC4A5, SLC4A2, SLCO2A1, SLC16A2	2.75E-03
PID	Integrins in angiogenesis	COL9A3, COL8A2, COL14A1, VEGFA, ADGRA2	3.54E-03
Reactome	Degradation of the extracellular matrix	COL9A3, COL8A2, COL14A1, COL18A1, HSPG2, FBN1	3.54E-03
Reactome	Extracellular matrix organization	COL9A3, COL8A2, COL14A1, COL18A1, HSPG2, FBN1, NID2, TTR	3.54E-03
Reactome	Retinoid cycle disease events	TTR, ABCA4, STRA6	3.54E-03
Reactome	Integrin cell surface interactions	COL9A3, COL8A2, COL18A1, HSPG2, FBN1	3.54E-03
NABA	Collagens	PCOL9A3, COL8A2, COL14A1, COL18A1	3.85E-03
Reactome	Collagen chain trimerization	COL9A3, COL8A2, COL14A1, COL18A1	3.85E-03
Reactome	Assembly of collagen fibrils and other multimeric structures	COL9A3, COL8A2, COL14A1, COL18A1	1.27E-02
Reactome	The canonical retinoid cycle in rods (twilight vision)	TTR, ABCA4, STRA6	1.28E-02
Reactome	Collagen degradation	COL9A3, COL8A2, COL14A1, COL18A1	1.28E-02
Reactome	Collagen biosynthesis and modifying enzymes	COL9A3, COL8A2, COL14A1, COL18A1	1.42E-02
Reactome	Response to elevated platelet cytosolic Ca2+	VEGFA, F5, ACTN2, IGF2, PHACTR2	1.47E-02
Reactome	Laminin interactions	COL18A1, HSPG2, NID2	2.16E-02
Reactome	Cargo concentration in the ER	F5, CD59, FOLR1	2.69E-02
Reactome	Transport of bile salts and organic acids, metal ions and amine compounds	SLC44A5, SLC31A1, SLC22A8, SLC13A4	2.86E-02
Reactome	Collagen formation	COL9A3, COL8A2, COL14A1, COL18A1	3.21E-02
NABA	Basement membranes	COL18A1, HSPG2, NID2	4.04E-02
Reactome	Visual phototransduction	HSPG2, TTR, ABCA4, STRA6	4.32E-02

*Pathway analysis of unique down-regulated genes altered by vehicle after TBI at 3 dpi in the hippocampus. Pathways were identified using a Fisher’s exact test with a false discovery rate of < 0.05.*

### Functional Interactive Network

**FIGURE 5:**
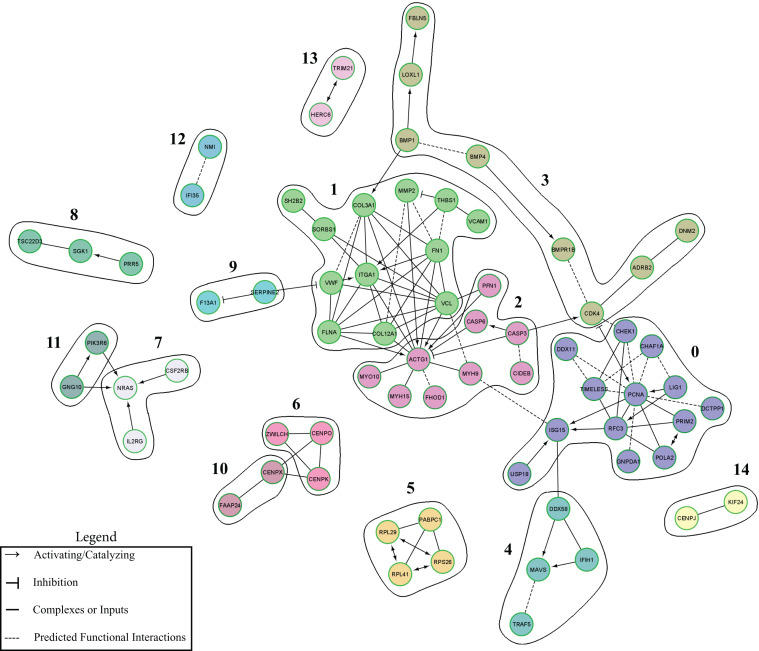
ReactomeFIViz-Derived Cluster Functional Interaction Network. Functional modules of unique up-regulated DEGs altered by candesartan treatment after TBI at 3 dpi in the hippocampus. Nodes in different network modules are shown in different colors. The modules listed are described in Supplementary Table 5.

A functional interactive network was created using an input of the 261 mapped DEGs that were uniquely up-regulated in the TBI + CD group at 3 dpi (Figure 5). The network included 71 nodes and 108 edges. Unlinked genes were not included in the network. The network was clustered into 15 functional modules. The genes with the highest degrees, included ACTG1 (Degree = 14), PCNA (Degree = 12), VCL (Degree = 10), ITGA1 (Degree = 9), FN1 (Degree = 8), RFC3 (Degree = 7), COL3A1 (Degree = 7), and FLNA (Degree = 7). Modules 0, 1, 2, and 3 had the greatest amount of nodes which were 13, 12, 9, and 8, respectively. Pathway enrichment was performed on the modules with 8 or greater nodes. The top enriched pathways were the following: Module 0- DNA replication, Telomere Maintenance, Synthesis of DNA, and S Phase. Module 1- Extracellular matrix organization, Beta1 integrin cell surface interactions, Integrin signaling pathway, and Focal adhesion. Module 2- Salmonella infection, Pathogenic E. coli infection, west nile virus, and apoptotic signaling in response to DNA damage. Module 3- Extracellular matrix organization, phospholipase c-epsilon pathway, cystic fibrosis transmembrane conductance regulator, and beta 2 adrenergic receptor pathway, and corticosteroids and cardioprotection (Supplementary Table 5).

### qPCR Confirmation

**FIGURE 6:**
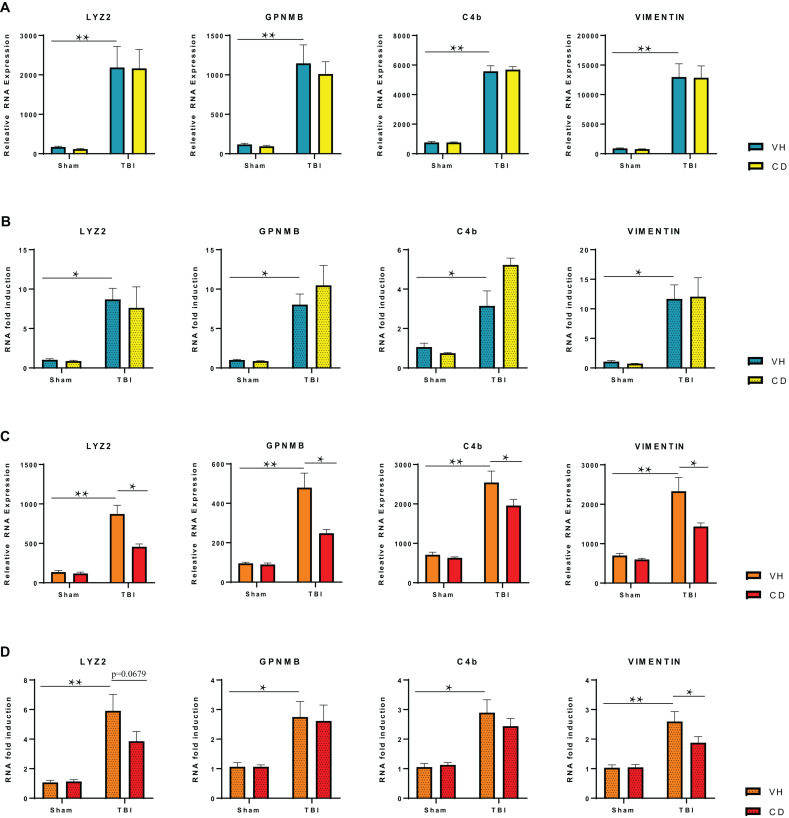
RT-qPCR Verification of Selected Differentially Expressed Genes in the Hippocampus at 3 and 29 dpi. **(A)** RNA-seq analysis shows highly elevated expression of specific genes in the ipsilateral hippocampus at 3 dpi that is not reduced on candesartan treatment. **(B)** RT-qPCR of RNA isolated from an independent cohort of injured mice confirmed changes seen in RNA-seq of injured hippocampi at 3 dpi. **(C)** RNA-seq analysis shows candesartan reduced the TBI mediated induction of RNA expression of these genes at 29 dpi. **(D)** RT-qPCR confirmed candesartan mediated reduction in expression of elevated gene Vimentin at 29 dpi, but not all investigated genes. C4b (complement C4B), Gpnmb (Glycoprotein Nmb), Lyz2 (Lysozyme 2) Vim (Vimentin) VH, vehicle; CD, candesartan. Mean +/– S.E.M., *n* = 4 **(A–C)**, *n* = 8 **(D)**. *p < 0.05, **p < 0.0001.

For confirmation of the RNA-seq data, we ran an additional cohort of mice through the identical experimental TBI and treatment paradigm as before. We isolated RNA and performed qPCR on four genes that the RNA seq data showed were elevated by TBI but were down-regulated by candesartan treatment at 29 dpi (Figure 6C). These genes are mainly expressed in microglia and astrocytes (Kamphuis et al., 2015; Kawahara et al., 2016; DePaula-Silva et al., 2019). All four genes were strongly up-regulated at 3 dpi in the hippocampus in both TBI + VH and TBI + CD groups. Candesartan treatment did not reduce expression of these genes at 3 dpi by either RNA seq or qPCR (Figures 6A,B). However, by 29 dpi, candesartan treatment reduced the expression of the genes Lyz2, C4b, and vimentin by both RNA seq and qPCR analysis (Figures 6C,D). GPNMB expression was reduced by candesartan in the samples that were analyzed by RNA-seq, but not by qPCR. Overall, the qPCR of an independent cohort of mice validated the RNA-seq analysis and indicated that the DEG analysis identified specific genes whose expression was reduced by candesartan treatment at 29dpi. Many of these genes, as the GO analysis showed (Table 3), encode proteins involved with immune and inflammatory processes (Supplementary Table 4).

## Discussion

This study focused on a transcriptomic analysis of individual mouse brain regions after TBI and after treatment with the angiotensin receptor blocker candesartan. We and others have previously shown that candesartan improves functional and morphological recovery in pre-clinical models of TBI (Villapol et al., 2012, 2015; Villapol and Saavedra, 2015). In this study we sought to understand the molecular mechanisms underpinning candesartan’s beneficial effects. Our data point to a role for candesartan in altering many different aspects of the response to TBI, particularly those involved with cellular response to stress, extracellular matrix alterations and the innate immune response. We found a more pronounced effect at an earlier (3 dpi) rather than a later time point (29 dpi) and in the area closer to the lesion (hippocampus) rather than further away (thalamus, hypothalamus). This transcriptomic analysis indicates several novel pathways that are altered by candesartan after brain injury that should assist in determining the molecular effects of candesartan’s beneficial actions in treating TBI.

The most pronounced differences within this transcriptomic analysis were the differences in gene expression between brain regions (Figures 2, 3). Surprisingly, these differences were greater than the differences after TBI at either time point or between candesartan and vehicle treated animals. This large difference in the transcriptome between the hippocampus, thalamus and hypothalamus indicates the very specialized functions that each of these brain regions has, and is similar to what others have found, in the mouse, and in higher organisms (Ng et al., 2009; Kasukawa et al., 2011; DiCarlo et al., 2017; Zhu et al., 2018). The effect of TBI on the transcriptome was most apparent in the hippocampus at 3 dpi, with over 1500 genes differentially expressed in the hippocampus after TBI in either vehicle or candesartan treated mice (Table 1). The pronounced effect of TBI within the hippocampus was expected given the proximity of the hippocampus to the location of the TBI. The effect of TBI on the transcriptome dissipated with distance from the lesion with a reduced effect in the thalamus and only one DEG in the hypothalamus at 3 dpi (Table 1). The TBI-induced differences in the transcriptome were much reduced at 29 dpi, with the hippocampus having the most DEGs at this later time point also.

We designed this study to identify candesartan-mediated changes in the brain transcriptome in response to TBI, in order to identify novel pathways through which candesartan could act to improve recovery from injury. Although the magnitude of the candesartan response was lower than expected, we did find many genes and pathways that were regulated by candesartan particularly in the hippocampus at 3dpi (Tables 2, 4 and Supplementary Table 1). Interferon signaling was one immune related pathway that was altered by candesartan at 3 dpi in the hippocampus. We found enrichment of genes involved with interferon signaling, including type 1 interferon (Table 4) at this early time point in the TBI + CD group. IFN signaling activates innate and adaptive immunity in response to infections and cell injury (Ivashkiv and Donlin, 2014). Activation of the type 1 IFN pathway in response to TBI induces neuro-inflammation, neuronal cell death, and glial reactivity (Karve et al., 2016). We observed up-regulation of the IFN signaling genes USP18 and ISG15 in the hippocampi of mice in the TBI + candesartan group at 3 dpi (Table 4). USP18 acts as a type 1 IFN receptor modulator, decreasing IFN responsiveness through blocking the interaction between JAK1 and the IFN receptor and therefore inhibiting downstream signaling events (Malakhova et al., 2006; Francois-Newton et al., 2011; Burkart et al., 2013; Wilmes et al., 2015; Honke et al., 2016; Basters et al., 2018). Induction of USP18 gene expression by candesartan would therefore inhibit interferon signaling in the cells in which this gene is expressed. ISG15 acts on USP18, stabilizing USP18 and preventing it from degradation. Induction of ISG15 by candesartan may therefore also reduce type 1 IFN signaling (Bihl et al., 2015). Some studies have shown that USP18 may act as a negative regulator of microglia activation through modulating IFNAR2, signaling, thus having a protective role on microglia function (Goldmann et al., 2015). Although both USP18 and ISG15 are both expressed in microglia, their expression in the brain is the strongest in endothelial cells (Zhang et al., 2014). Thus, candesartan may be interfering with interferon signaling in microglia and endothelial cells after TBI.

Pathway analysis of the effects of candesartan at 3 dpi in the hippocampus identified nine genes associated with chromosome maintenance and other processes associated with DNA replication (Table 4). These were also part of the reactome (Figure 5). The majority of these genes encode non-specific regulators of DNA synthesis. Angiotensin II has been shown to promote DNA replication in vascular smooth muscle and other cell types (Makita et al., 1995; Pawlikowski et al., 1999; Touyz et al., 1999; Chiu et al., 2003) and candesartan can mitigate angiotensin II induced DNA damage (Schmid et al., 2008). DNA replication and repair are important components of the response to TBI, with problems in DNA repair, and enduring DNA damage contributing to functional deficits after TBI (Davis and Vemuganti, 2020). Candesartan’s actions in regulating DNA repair and synthesis may indicate an additional pathway through which it has beneficial action after TBI.

Both GO (Table 2) and pathway analysis (Table 4) identified multiple genes associated with blood vessel and circulatory system development up-regulated in the hippocampi of the TBI + CD mice, suggesting a possible role for candesartan in the regulation of vascular endothelial cells within the hippocampus after injury. Additionally, pathway analysis of down-regulated unique genes in the TBI + VH condition identified a reduction in integrins of angiogenesis (Table 5). AT1 receptor blockade utilizing candesartan stimulates angiogenesis through the regulation of vascular endothelial factor (VEGF) and offers protection in animal models of ischemic retinopathy (Shanab et al., 2015) and stroke (Guan et al., 2011). There are many adverse effects of TBI on the cerebral vasculature, including edema and chronic inflammation (Salehi et al., 2017). Candesartan mediated improvements in angiogenesis and vascular function after TBI are an obvious target as the AT1 receptor is expressed throughout the vasculature. These transcriptomic data suggest that candesartan is functioning through such mechanisms after TBI.

Genes associated with the extracellular matrix (ECM) were also highly represented in our analysis of candesartan activity at 3dpi in the hippocampus. Candesartan administration after TBI resulted in the up-regulation of GO families associated with cell adhesion (Table 2), and pathway analysis identified the ECM organization as the most significantly up-regulated reactome (FDR < 0.05) (Table 4). The angiotensin system’s modulation of the ECM has been studied extensively in its role in cardiac disease (Weber, 1997; Singh et al., 2008; Dab et al., 2012) and suggested to play a role in neural plasticity (Wright and Harding, 2004). The maintenance of the ECM plays an important role in modulating inflammation and is a potentially important factor in repair after TBI (Gaudet and Popovich, 2014).

Candesartan treatment for 3 days in sham injured mice resulted in significant differential gene expression (FDR 0.05, abs log2 FC > 0.32) in the hippocampus relative to that in sham injured mice treated with vehicle (Table 1). Interestingly, this effect was not noted in any of the other brain regions suggesting that this effect is hippocampal-specific. As these mice received a skin incision with isoflurane administration without a craniotomy it is surprising that this differential expression is not seen in either the hypothalamus or the thalamus, particularly given the high level of expression of AT1R in the hypothalamus (Nishimura et al., 2000; de Kloet et al., 2015; Wang et al., 2016b). These data suggest therefore that either candesartan has greater access to the hippocampus, than the thalamus or hypothalamus, perhaps indicating region specific differences in the blood brain barrier; or that candesartan has different effects within specific brain regions. Analysis of the candesartan effects in the hippocampus of sham mice shared some gene ontology categories with those from unique DEGs identified in the hippocampus of mice treated with candesartan after TBI. Specifically, these included genes involved with cilium organization and structure, and ion transport (Table 2 and Supplementary Table 2). To our knowledge this is the first report of candesartan-specific gene regulation in the hippocampus of mice without brain injury. As several different behavioral effects have been noted for candesartan and related ARBs (Wright and Harding, 2004; Khoury et al., 2012; Nylocks et al., 2015), our data provide useful information as to their potential effects in specific brain regions. This hippocampal-specific effect of candesartan in sham mice was no longer detectable in mice treated for 29 dpi. It is possible that longer term treatment led to desensitization of receptors or other signaling molecules to reduce this effect, particularly as the 29 dpi mice had candesartan administered through an osmotic minipump, providing constant low dose administration in contrast to the daily injections received by the 3 dpi group mice.

The overall effect of candesartan treatment in mice after TBI was smaller than expected. We used a sub-hypotensive dose of candesartan (0.1 mg/kg/day) that we and others have previously shown to be effective for improving recovery from CCI in mice (Timaru-Kast et al., 2012; Villapol et al., 2012, 2013, 2015). However, this low dose did not produce robust changes in gene expression, even in the hippocampus. The changes in gene expression with candesartan treatment after TBI were swamped by those produced in response to the TBI, particularly in the hippocampus. Thus, while there were 1732 DEGs in the hippocampus in the TBI + CD group in comparison to the Sham + VH group, 1166 of these genes were also found in the TBI + VH group. Nonetheless, candesartan treatment did produce important changes in gene expression in specific pathways, even at the acute 3 dpi time point. The magnitude of these changes were not as great as those produced by TBI. However, as this dose of candesartan has therapeutic efficacy in this model of TBI, it is possible that these smaller changes in gene expression, or those that were more variable in our hands, may be relevant to its mechanism of action.

An additional explanation for the relatively small effects of candesartan on gene expression after TBI, is the potentially small effect of blocking the AT1 angiotensin receptor after injury. The number of cells that have high level expression of this receptor in the hippocampus and thalamus is quite small (Lenkei et al., 1997, 1998), and the effect of angiotensin II after TBI is not known. Examination of gene expression changes in specific cell types that are known to express the AT1R, such as endothelial cells and neurons, may have produced stronger gene expression changes. In the present study, some significant cell-specific alterations in gene expression by candesartan may have been masked by unchanged expression in other cell types. We have previously shown that AT1aR KO mice are partially protected from TBI, with a smaller lesion and reduced GFAP expression after injury (Villapol et al., 2015), suggesting that signaling through the AT1R does play some role in the response to TBI in mice. Additionally, candesartan efficacy in improving recovery in a mouse model of TBI is partially also due to its partial agonism of the PPARγ receptor (Villapol et al., 2012, 2015). As PPARγ is more widely expressed than the AT1R (Bernardo and Minghetti, 2006), stimulating PPARγ receptor signaling expands the cellular repertoire of candesartan. However, in this study we were not able to differentiate between expression changes that were a result of antagonism of the AT1R or agonism of PPARγ.

Another limitation of our study was that our sham group of animals was anesthetized with a skin incision only without craniectomy. It has been shown that even a very careful craniectomy can cause minor damage to the brain parenchyma, simulating a mild brain injury (Cole et al., 2011; Lagraoui et al., 2012). Therefore, many groups, including ours, have switched to using mice without a craniectomy as a sham. Our original studies that showed candesartan efficacy in the mouse CCI model were run using this sham (Villapol and Saavedra, 2015; Villapol et al., 2015), and as we replicated those studies here, we used the same controls. Nonetheless, there are limitations in comparing the transcriptome in brain regions after craniectomy and TBI with those in a brain without either. Potential damage to the bone or meninges could influence gene expression in the underlying brain. As the brain regions investigated here do not lie immediately below the skull, this may be less of a concern. A potential alternate control could have been the contralateral, uninjured corresponding brain region. However, it has also been shown that the contralateral side is not completely unaltered by the injury (Rola et al., 2006; Urrea et al., 2007), and the contralateral regions were not considered good controls for the comparisons with the injured brain run here. We therefore feel that the sham injured animals we used as controls were the most appropriate for our specific experiments, although there are limitations associated with their use.

We and others have previously shown that administration of ARBs after brain injury has anti-inflammatory activity, including reduction of reactive astrocytosis and microgliosis, and reduction of cytokines in the peri-lesional area (Timaru-Kast et al., 2012; Villapol et al., 2012, 2015) even at acute times after injury. However, in this study candesartan administration did not reduce TBI-induced expression of multiple pro-inflammatory genes at 3 dpi in the hippocampus (Figure 6 and data not shown). We interpret these data to show the considerable strength of the TBI-mediated induction of inflammatory gene expression at this early time point. This discrepancy in candesartan action with similar dose and route of administration, between our prior findings, and those of this transcriptomic analysis is likely not explainable by the difference between the induction of RNA, in this paper, and protein in our prior publications. However, the anti-inflammatory action of candesartan was detectable in the RNA seq data from the hippocampi of mice at 29 dpi where candesartan did reduce expression of several inflammatory genes and genes associated with reactive gliosis including GFAP (Figure 6 and Supplementary Table 4). This action was also reflected in the GO analysis of unique genes that were up-regulated in the TBI + VH group but not in the TBI + CD group at 29dpi in the hippocampus. These GO terms include immune response, regulation of the immune response, and inflammatory response amongst other terms (Table 3).

The reduction in inflammatory gene expression at 29 dpi is probably the result of candesartan action on several different cell types, with an important microglial component. Interestingly, we observed that some genes associated with the transcriptomic signature for Disease Associated Microglia (DAM) were shown in our unique gene analysis at 29 dpi in the hippocampus (Keren-Shaul et al., 2017; Deczkowska et al., 2018). These genes, including Trem2, Itgax and Spp1 were upregulated only in the TBI + VH group, implying that candesartan reduced their expression. There were many mainly microglial-specific genes, associated with the unique gene analysis in the TBI + VH group at 29dpi, including Clq, Tlr7, Itgb2, C3ar1 and Csfr1 adding to the evidence that candesartan had a specific effect on activated microglia at this time point (Zhang et al., 2014). One gene we identified as being reduced by candesartan treatment after TBI at 29dpi in the hippocampus was the Lyz2 gene, despite this gene not being part of the unique gene analysis at this time point. The Lyz2 gene encodes the lysosomal enzyme lysozyme M (Orthgiess et al., 2016) which in mice is highly expressed within peripheral macrophages and monocytes (Cross et al., 1988), and whose promoter use in LysM-Cre mice has driven many macrophage-specific mouse knockouts (Dosch et al., 2019). Although once thought of as a microglial gene, Lyz2 now forms part of the transcriptomic signature for infiltrating monocytes into the CNS (Izzy et al., 2019; Ronning et al., 2019; Ellwanger et al., 2021), so its reduction by candesartan may indicate reduced infiltration of these monocytes at this later time point. A closer examination of the effect of candesartan treatment on microglial and macrophage lineage cells in the CNS after injury will require cell sorting and scRNA seq analysis on these different cellular populations.

Although the effects of candesartan in the hippocampus could be mediated by either/both antagonism at the AT1R or agonism at the PPARγ receptor, it is also possible that a third receptor mediates some of the changes in gene expression specifically in the hippocampus. The Mas receptor is very highly expressed in the dentate gyrus of the hippocampus (Freund et al., 2012), and our RNA seq data indicate that at least at the level of RNA, is much more highly expressed than the AT1a receptor in the hippocampus (Supplementary Figure 3). As candesartan has been shown to induce activity and expression of ACE2, the enzyme that converts Ang II to the Mas receptor ligand, Ang-(1-7), it has been postulated that some of candesartan’s beneficial effects may be mediated by enhancing activation of the ACE2/Ang-(1-7)/MasR axis (Pernomian et al., 2015). Indeed, we have previously shown that Ang-(1-7) treatment after TBI can also enhance recovery (Janatpour et al., 2019). Further delineation of which receptor mediates candesartan’s regulation of gene expression will await experiments involving specific receptor knockout mice.

The data we present here provide an array of information on the response to TBI in different brain regions at different time points, and the effects of candesartan on these responses. We have shown that candesartan can alter multiple pathways including interferon signaling, extracellular matrix alterations, DNA replication and manipulation of cerebrovascular repair and function. Some of these functions are in agreement with a prior microarray study of candesartan action on primary cerebellar granular neurons in response to glutamate treatment (Elkahloun et al., 2016), suggesting that candesartan can act directly on neurons in the intact brain. However, we also propose that much of the beneficial effects of candesartan after TBI will be on glia and the cerebrovasculature. This study will serve as a starting point for a more detailed and granular examination of candesartan action on specific cell types, and pathways to delineate the molecular actions of this drug after TBI. Further understanding of the complex interactions through which candesartan can improve the pathophysiology of TBI may help provide future targets for treatment.

## Data Availability Statement

The datasets presented in this study can be found in online repositories. The names of the repository/repositories and accession number(s) can be found below: GEO database, GSE163415.

## Ethics Statement

The animal study was reviewed and approved by Uniformed Services University Institutional Animal Care and Use Committee.

## Author Contributions

PA, CD, and AJS planned the experiments. PA, MR, and AI performed the experiments. PA, AS, MW, and DS analyzed the data. PA, MW, AS, CD, DS, and AJS interpreted the analysis. PA, DS, and AJS wrote the manuscript. All authors contributed to the article and approved the submitted version.

## Conflict of Interest

The authors declare that the research was conducted in the absence of any commercial or financial relationships that could be construed as a potential conflict of interest.

## Footnotes

1 http://www.GeneOntology.org

2 http://www.gsea-msigdb.org

3 http://www.biocarta.com

4 http://www.genome.ad.jp

5 http://www.ndexbio.org

6 http://www.reactome.org
