# Supplementary figures and images for: Transcriptomic Analysis of Mouse Brain After Traumatic Brain Injury Reveals That the Angiotensin Receptor Blocker Candesartan Acts Through Novel Pathways
Source: Front Neurosci. 2021 Mar 22;15:636259. doi: 10.3389/fnins.2021.636259 (PMC8019829; doi:10.3389/fnins.2021.636259)

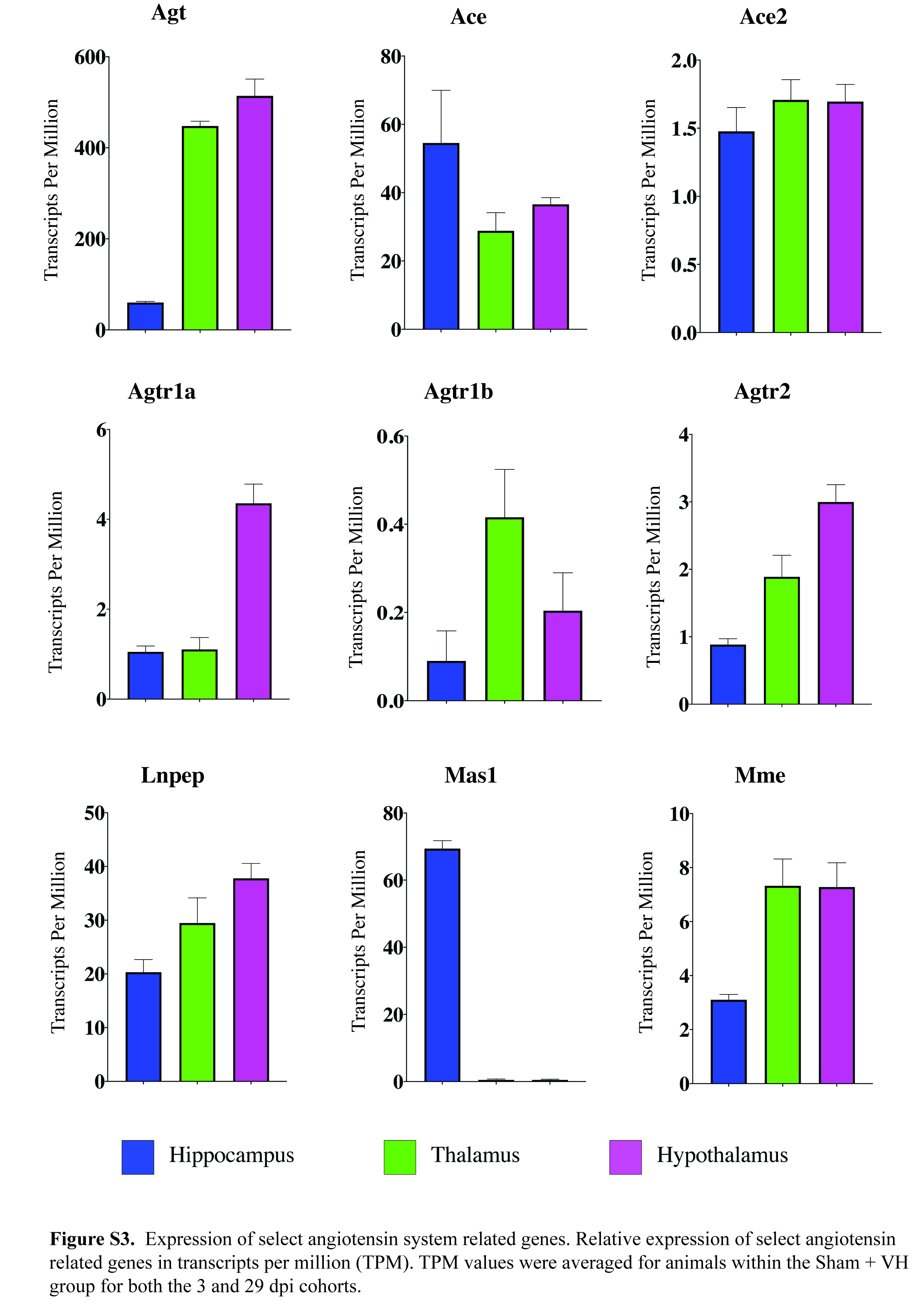

Supplement: Supplementary file 8 [file Image_3.TIF]
